# Beyond Theory of Mind: mentalization as a relational and developmental framework for autism

**DOI:** 10.3389/fpsyt.2026.1879738

**Published:** 2026-07-15

**Authors:** Assia Riccioni, Cristina Di Vincenzo, Alessia Sabina De Frenza, Carola Leone, Ilaria Bertoncini, Lucrezia Arturi, Luigi Mazzone, Stefano Vicari, Maria Pontillo

**Affiliations:** 1 Child Neurology and Psychiatry Unit, Department of Mental and Neurological, Dental and Sensory Organ Health, Policlinico Tor Vergata University Hospital, Rome, Italy; 2 Department of Psychology, Università Cattolica del Sacro Cuore, Milan, Italy; 3 Child and Adolescent Neuropsychiatry Unit, Bambino Gesù Children’s Hospital, IRCCS, Rome, Italy; 4 Systems Medicine Department, University of Rome Tor Vergata, Rome, Italy; 5 Life Sciences and Public Health Department, Catholic University of the Sacred Heart, Rome, Italy

**Keywords:** autism, co-occurring psychopathology, mentalization, mentalization-based treatment, social cognition, Theory of Mind

## Abstract

Autistic individuals and those around them often navigate social and emotional situations in which behaviors, intentions, and affects are difficult to interpret. Supporting mentalizing processes within child–caregiver interactions may help address these challenges; however, a broader conceptual shift is needed, moving beyond a narrow deficit-based perspective toward understanding mentalization as a multidimensional, relational, and developmental process. By shifting the focus from individual deficits to child–caregiver meaning-making processes, this framework may help clarify assessment and intervention targets and inform future research on psychopathological vulnerability in autism. This targeted narrative mini-review therefore aimed to summarize preliminary evidence suggests that other-related mentalizing may show greater difficulties than self-related mentalizing, although this hypothesis requires further replication. Findings also highlight caregiver mentalization, particularly parental reflective functioning, as a key relational process shaping how children’s behavior is interpreted, regulated, and responded to over time. In this light, preliminary intervention studies suggest that mentalization-based and mentalization-informed approaches may improve parental reflective functioning, cognitive reappraisal, self-efficacy, and inferential style, with potential indirect benefits for children’s emotional outcomes. We therefore propose a relational-developmental framework in which mentalization is conceptualized as a shared and dynamic process of meaning-making under conditions of social and emotional ambiguity. Adopting an individual, relational and developmentally informed perspective may contribute to the development of more precise assessment models, more targeted interventions, and a deeper understanding of mental health vulnerability in autism.

## 1 Introduction

Mentalization broadly refers to the capacity to understand one’s own and others’ behavior in terms of intentional mental states, such as thoughts, feelings, desires, and beliefs (1, 2). It can be conceptualized as a form of imaginative mental activity that allows individuals to interpret human behavior as meaningful and psychologically driven. In autism research, difficulties in understanding mental states have historically been approached mainly through the lens of Theory of Mind (ToM), a construct referring to the ability to attribute mental states to oneself and others and to use this information to predict behavior. Since the seminal work of Baron-Cohen and colleagues, ToM impairment has been considered a key explanatory account of social-communicative difficulties in autism (3, 4). As a consequence, ToM has become an important target for intervention, informing psychoeducational and therapeutic approaches designed to enhance social understanding in autistic children and adolescents (5, 6).

However, reducing mentalization to ToM may obscure the broader clinical and developmental processes through which autistic individuals, their caregivers, and clinicians interpret behavior, emotions, and interpersonal experiences. Compared with ToM, mentalization represents a broader and more multidimensional construct, encompassing not only explicit reasoning about beliefs and intentions, but also implicit, affective, self-related, other-related, and relational processes (1, 2, 7). In the present mini-review, mentalization is conceptualized as an overarching framework encompassing multiple dimensions of understanding mental states, including self- and other-related, cognitive and affective, implicit and explicit, as well as relational processes. Related constructs such as ToM, social cognition, metacognition, and reflective functioning are considered partially overlapping yet conceptually distinct domains that capture specific aspects of this broader mentalizing framework. We propose that this distinction is particularly relevant in autism, as reducing mentalization to ToM risks framing autism through a narrow deficit-based perspective centered on the individual child’s difficulty in understanding others’ mental states. Conversely, conceptualizing mentalization as a multidimensional and relational process may provide a more clinically meaningful framework for understanding heterogeneity in social functioning, caregiver–child interactions, emotion regulation, and vulnerability to co-occurring psychopathology in autism.

This perspective is especially important given the high prevalence of co-occurring psychiatric conditions in autism and the established association between mentalizing difficulties and several forms of psychopathology, including affective, anxiety, and psychotic disorders (2, 8–11).

From a clinical perspective, mentalization-based approaches have been increasingly applied to emotional dysregulation and interpersonal difficulties across a range of psychiatric conditions (12). More recently, mentalization-informed approaches have begun to be adapted for autistic individuals, with the aim of supporting social understanding, affect regulation, and relational functioning (7). Yet, their application in autism remains complicated by the lack of a shared and coherent definition of mentalization within this field. Despite its clinical relevance, the term “mentalization” in autism continues to be used ambiguously and inconsistently, frequently overlapping with related constructs such as ToM, social cognition, metacognition, and reflective functioning. This lack of terminological precision contributes to substantial ambiguity in both research and clinical practice, limiting the comparability of findings, complicating the operationalization of the construct, and making it difficult to identify specific treatment targets. Importantly, this fragmentation is not merely a methodological limitation; rather, it reflects the need to better organize mentalization across clinically meaningful dimensions, including self- versus other-related processes, individual versus relational levels, and explicit versus implicit forms of mentalizing.

This conceptual ambiguity has at least two major consequences. First, it limits the possibility of defining a clear mentalizing profile in autism, including the extent to which difficulties involve not only understanding one’s own and others’ mental states, but also the dynamic relationship between the two. Consequently, autism may continue to be framed through broad notions of generalized “*mindreading deficits*”, rather than through specific and developmentally differentiated profiles of mentalization. Second, this ambiguity has direct clinical implications. Without a clearer definition of which mentalizing components are affected, interventions may lack precise targets, making it more difficult to develop treatments that support emotional regulation, social understanding, and adaptive functioning across social, educational, and occupational contexts.

Considering these issues, the present mini-review aims to examine mentalization in autistic children and adolescents by moving beyond a narrow deficit-based account and proposing a relational and developmental framework. Specifically, we first discuss why mentalization should be understood as a multidimensional construct rather than a unitary impairment. Second, we examine how mentalization operates across individual, relational and developmental levels. Finally, we discuss how this framework may inform mentalization-based interventions and guide future research on psychopathological vulnerability in autism.

## 2 Methods

This mini-review was conducted as a targeted narrative review aimed at examining how mentalization has been conceptualized, operationalized, and clinically applied in autism research, with a particular focus on autistic children and adolescents and caregiver-child processes. Given the conceptual and integrative aim of the article, a formal systematic review methodology was not adopted.

Relevant literature was identified through targeted searches in PubMed and manual screening of the reference lists of key articles. Searches focused on autism and mentalization-related constructs, including mentalization, reflective functioning, parental reflective functioning, metacognition, social cognition, and mentalization-based or mentalization-informed interventions. Searches were limited to English-language publications and were conducted up to February 2026.

Studies were selected on the basis of their relevance to the aims of the review. Eligible empirical studies included cross-sectional, longitudinal, and intervention designs examining mentalization, reflective functioning, or related constructs in autistic children and adolescents (up to 18 years of age) and/or their caregivers. Caregiver-focused studies were included because they were directly relevant to the relational dimension of the proposed framework. Theoretical papers, clinical papers, reviews, and study protocols were not treated as empirical evidence but were considered, when relevant, for the conceptual discussion.

Findings were synthesized narratively and organized into three domains: (1) differentiated mentalizing profiles in autistic children and adolescents, (2) parental reflective functioning and caregiver-child mentalizing processes, and (3) mentalization-based or mentalization-informed interventions. The synthesis aimed to identify areas of convergence and divergence and to develop a relational-developmental framework for future research and clinical application.

## 3 Results

### 3.1 From mentalizing deficits to differentiated mentalizing profiles

A mentalization perspective allows for a more differentiated view, in which mentalizing difficulties may vary across distinct components, including self- versus other-related processes, explicit versus implicit mentalizing, and cognitive versus affective dimensions. This distinction is clinically important because it shifts the focus from whether autistic individuals “lack” mentalization to which aspects of mentalizing are vulnerable, preserved, or context dependent.

This distinction becomes particularly relevant when self- and other-related mentalizing are examined separately. Amodeo et al. (13) investigated different components of mentalization in 30 autistic adolescents and 26 neurotypical controls, matched for age and IQ. Mentalization was defined as the ability to monitor and interpret one’s own and others’ mental states and was operationalized through a Feeling-of-Knowing task for self-related mentalizing and the Frith-Happé animations for other-related mentalizing. Autistic adolescents showed significantly reduced performance in other-related mentalizing (p = 0.028), whereas no group differences emerged in self-related mentalizing or self-bias measures (all p > 0.05). These findings suggest that mentalization difficulties in autism may not reflect a generalized impairment, but rather a differentiated profile in which other-related mentalizing is more vulnerable than self-related processes, with self-related mentalizing and self-bias remaining relatively preserved.

These findings suggest the possibility of a differentiated mentalizing profile in autism, with reduced performance observed in other-related mentalizing and no detectable group differences in self-related mentalizing. However, this interpretation currently relies on a single study and requires further replication.

### 3.2 Mentalization as a caregiver-child regulatory system

A second source of complexity in defining mentalization in autism concerns the level of analysis at which the construct is examined. While mentalization is often conceptualized as an individual capacity, autism research increasingly suggests that mentalizing processes should also be understood within caregiver-child relationships. In this context, parental reflective functioning (PRF) represents a relational expression of mentalization, referring to the caregiver’s capacity to interpret the child’s behavior in terms of underlying mental states. This perspective is particularly relevant in autism, where caregivers are often required to make sense of behaviors that may be difficult to interpret, emotionally intense, or socially ambiguous.

Enav et al. (14) examined whether parental reflective functioning (PRF) moderated the association between child maladaptive behaviors and parental hopelessness in a cohort of 68 parents of autistic children aged 3–18 years. Notably, this report was based on the same sample previously described by Enav et al. (15), although it addressed a different research question. PRF was defined as the caregiver’s capacity to understand and interpret the child’s internal states. Higher levels of child maladaptive behaviors were associated with greater parental hopelessness (p < 0.05), and PRF significantly moderated this association (p < 0.05), with stronger effects among parents with lower reflective functioning. These findings suggest that caregiver mentalization may buffer the psychological impact of child difficulties by supporting more flexible and contextualized interpretations of behavior.

Further evidence comes from Enav et al. (16), who investigated PRF in 30 families with one autistic child and one typically developing sibling, and 30 families with only typically developing children, for a total of 120 child-level observations. PRF was assessed using both the Parental Reflective Functioning Questionnaire and the Reflective Functioning Scale applied to the Five-Minute Speech Sample. Parents showed higher reflective functioning toward autistic children (p < 0.001), together with greater interest and curiosity (p < 0.05), but also higher levels of pre-mentalizing modes (p < 0.001). This pattern suggests that caregiving in autism may require a more effortful reflective stance, which can coexist with moments of uncertainty, rigidity, or difficulty in interpreting the child’s mental states.

Longitudinal findings further support the view of mentalization as a dynamic caregiver-child process. Zhang et al. (17) examined reciprocal associations between PRF and behavioral problems in 180 parents of autistic children across three time points over six months. Higher parental pre-mentalizing predicted subsequent increases in children’s internalizing problems (p < 0.05), while greater child difficulties predicted later reductions in adaptive parental reflective functioning, including lower certainty and curiosity about mental states (p < 0.05). These bidirectional associations suggest that mentalization in autism is not only a stable parental characteristic, but part of an ongoing transactional process between child functioning and caregiver responses.

Taken together, these findings suggest that conceptualizing mentalization in autism solely as an individual ability may be insufficient. However, the current evidence base remains limited, with several studies originating from the same research program. Further replication by independent groups is needed to establish the robustness and generalizability of these observations. Mentalization may also operate at the relational level, shaping how caregivers interpret, regulate, and respond to autistic children’s behavior.

### 3.3 Mentalization-based interventions: targeting caregiver-child processes

The relational nature of mentalization has direct implications for intervention. If caregiver mentalization shapes how children’s behaviors are interpreted, regulated, and responded to, then mentalization-based approaches may be particularly relevant for supporting caregiver-child interactions in autism. In this context, interventions do not simply aim to increase parental knowledge about autism, but to promote a more flexible and reflective stance toward the child’s behavior and emotional experience. However, the current evidence base remains preliminary and is still largely focused on caregiver-oriented interventions.

Enav et al. (15) evaluated the efficacy of a brief mentalization-based group intervention in 68 parents of autistic children aged 3 to 18 years. The intervention consisted of weekly 90-minute sessions focusing on emotion regulation and reflective understanding of parent–child interactions, with mentalization targeted through the enhancement of parental reflective functioning. Results showed significant changes in the intervention group (p = 0.0002), compared with no change in the delayed-treatment group (p = 0.72). Parents also reported stronger beliefs in the malleability of emotions (p = 0.002), greater parental self-efficacy, and reductions in children’s internalizing symptoms (p = 0.01). These findings suggest that mentalization-based interventions may support caregivers’ reflective and regulatory capacities while also producing indirect benefits for child emotional outcomes. Building on this intervention work, Enav et al. (18) examined a potential mechanism of change by testing whether a 4-week reflective parenting workshop could support cognitive reappraisal in 27 parents of autistic children aged 3 to 18 years. Mentalization was operationalized as reflective reappraisal, defined as the reinterpretation of emotional situations through consideration of one’s own and others’ mental states. Results showed increased cognitive reappraisal following the intervention, both when assessed through the Emotion Regulation Questionnaire (p < 0.05) and through narrative ratings (p < 0.001). Exploratory analyses further showed a significant time × reappraisal type interaction (p < 0.01), indicating that the observed changes were mainly driven by reflective reappraisal, whereas non-reflective reappraisal did not significantly change. These findings suggest that mentalization-based interventions may support adaptive emotion regulation by fostering mental-state-oriented reinterpretation processes. Similarly, Parashar et al. (19) examined the effects of a brief reflective parenting intervention in 13 parents of young autistic children. Mentalization was conceptualized as reflective processing of emotional and interpersonal experiences. Results showed a significant reduction in negative inferential style from pre- to post-intervention (p = 0.001). These findings indicate that mentalization-informed interventions may be beneficial even in brief and pragmatic formats, supporting more reflective interpretations of emotionally salient experiences. Overall, these studies suggest that mentalization-based interventions in autism are promising but still preliminary. Available evidence mainly concerns caregiver-oriented approaches. Positive changes have been reported in parental reflective functioning, cognitive reappraisal, parental self-efficacy, and inferential style. Child outcomes appear to be mostly indirect, particularly through reductions in internalizing symptoms. Together, these findings suggest that mentalization in autism cannot be reduced to a single individual ability, but should be organized across distinct yet interconnected levels. The relational nature of mentalization has direct implications for intervention. If caregiver mentalization shapes how children’s behaviors are interpreted, regulated, and responded to, then mentalization-based approaches may be particularly relevant for supporting caregiver-child interactions in autism. In this context, interventions do not simply aim to increase parental knowledge about autism, but to promote a more flexible and reflective stance toward the child’s behavior and emotional experience. However, the current evidence base remains preliminary and is still largely focused on caregiver-oriented interventions.

Enav et al. (15) evaluated the efficacy of a brief mentalization-based group intervention in 68 parents of autistic children aged 3 to 18 years. The intervention consisted of weekly 90-minute sessions focusing on emotion regulation and reflective understanding of parent–child interactions, with mentalization targeted through the enhancement of parental reflective functioning. Results showed significant improvement in the intervention group (p = 0.0002), compared with no change in the delayed-treatment group (p = 0.72). Parents also reported increased beliefs in the malleability of emotions (p = 0.002), greater parental self-efficacy, and reductions in children’s internalizing symptoms (p = 0.01). These findings suggest that mentalization-based interventions may improve caregiver functioning while also producing indirect benefits for child emotional outcomes.

Building on this intervention work, Enav et al. (18) examined a potential mechanism of change by testing whether a 4-week reflective parenting workshop could improve cognitive reappraisal in 27 parents of autistic children aged 3 to 18 years. Mentalization was operationalized as reflective reappraisal, defined as the reinterpretation of emotional situations through consideration of one’s own and others’ mental states. Results showed increased cognitive reappraisal following the intervention, both when assessed through the Emotion Regulation Questionnaire (p < 0.05) and through narrative ratings (p < 0.001). Exploratory analyses further showed a significant time × reappraisal type interaction (p < 0.01), indicating that improvement was mainly driven by reflective reappraisal, whereas non-reflective reappraisal did not significantly change. These findings suggest that mentalization-based interventions may support adaptive emotion regulation by specifically strengthening mental-state-oriented reinterpretation processes.

Similarly, Parashar et al. (19) examined the effects of a brief reflective parenting intervention in 13 parents of young autistic children. Mentalization was conceptualized as reflective processing of emotional and interpersonal experiences. Results showed a significant reduction in negative inferential style from pre- to post-intervention (p= 0.001). These findings indicate that mentalization-informed interventions may be effective even in brief and pragmatic formats, improving how caregivers interpret emotionally salient events.

Overall, these studies suggest that mentalization-based interventions in autism may represent a promising area of investigation. However, the evidence base remains preliminary and is currently concentrated within a small number of research groups, highlighting the need for independent replication and larger controlled studies.

These findings suggest that mentalization in autism cannot be reduced to a single individual ability, but should be organized across distinct yet interconnected levels. A summary of these levels and their contribution to the proposed framework is provided in **Table 1**.

**Table 1:** Levels of mentalization in autism and contribution to the proposed framework.

Conceptual domain	Core mentalization process	Supporting evidence	Contribution to the proposed framework
Individual	Differentiated self- and other-related mentalizing	Amodeo et al. (13)	Preliminary evidence of reduced other-related mentalizing, with no detectable differences in self-related mentalizing.
Relational	Caregiver interpretation of the child’s behavior	Enav et al. (14)Enav et al. (16)	Shift from child-only deficits to caregiver–child mentalizing processes
Developmental	Bidirectional caregiver–child influences over time	Zhang et al. (17)	Preliminary evidence for transactional developmental processes
Intervention	Reflective parenting and reflective reappraisal	Enav et al. (15)Enav et al. (18)Parashar et al. (19)	Caregiver mentalization, interpretative flexibility, and emotion regulation as intervention targets

## 4 Discussion

The present mini-review highlights that mentalization could represent a clinically and theoretically relevant construct in autism, yet its conceptualization remains fragmented and inconsistently defined across research domains. Although difficulties in understanding mental states have long been considered central to autism, the evidence reviewed suggests that mentalization should not be reduced to a global impairment in ToM. Rather, mentalization appears to involve differentiated and context-sensitive processes that operate across individual, caregiver-child, and developmental levels. As a consequence, the study of mentalization in autism requires a shift from an individual, deficit-based model to a relational and developmental framework.

At the individual level, available findings suggest that mentalizing difficulties in autism may not be uniform across domains.

Evidence of reduced other-related mentalizing alongside no detectable differences in self-related mentalizing suggests the possibility that specific components of mentalization may be differentially affected (13). However, this interpretation currently relies on a limited evidence base and requires replication across independent studies. In addition, this pattern challenges unitary accounts of mentalization and supports the need to distinguish among self- and other-related processes, as well as between broader mentalization and related constructs such as ToM, social cognition, metacognition, and reflective functioning. Importantly, many traditional ToM paradigms primarily assess explicit and cognitively mediated forms of mental-state reasoning, which may only partially capture the affective and relational dimensions of mentalization relevant to everyday social functioning.

From this perspective, the key question is not whether autistic children and adolescents can or cannot mentalize, but which aspects of mentalizing may be more or less affected, and under which contextual conditions. This distinction is particularly relevant for understanding and potentially preventing co-occurring psychopathological conditions, which are known to occur at increased rates in autistic individuals (8) and are often influenced not only by individual vulnerability, but also by relational and contextual factors (20).

At the relational level, the findings reviewed highlight the importance of caregiver mentalization, particularly parental reflective functioning. Studies on parents of autistic children suggest that the caregiver’s capacity to interpret the child’s behavior in terms of mental states may buffer the psychological impact of child difficulties, reduce hopelessness, and support more flexible responses to challenging behaviors (14, 16). This is particularly relevant in autism, where caregivers may often be required to make sense of behaviors that are ambiguous, emotionally intense, or difficult to interpret. In this light, the clinical task may involve what Slade (21) described as “mentalizing the unmentalizable”: helping caregivers remain curious and reflective even when the child’s behavior appears difficult to understand, non-reciprocal, or difficult to connect with emotionally.

This relational perspective is also consistent with broader accounts suggesting that social difficulties in autism may arise not only from individual impairments, but also from reciprocal mismatches in understanding between autistic and non-autistic individuals (22). Although this perspective comes from a different theoretical tradition, it is useful for the present discussion because it cautions against locating mentalizing difficulties exclusively within the autistic child. Instead, it supports a more systemic view in which meaning-making, misunderstanding, and emotional regulation emerge within interactions. Mentalization may therefore be understood not simply as an individual social-cognitive skill, but as a relational process through which children, caregivers, and clinicians attempt to construct shared meaning under conditions of social and emotional ambiguity.

**Figure 1:**
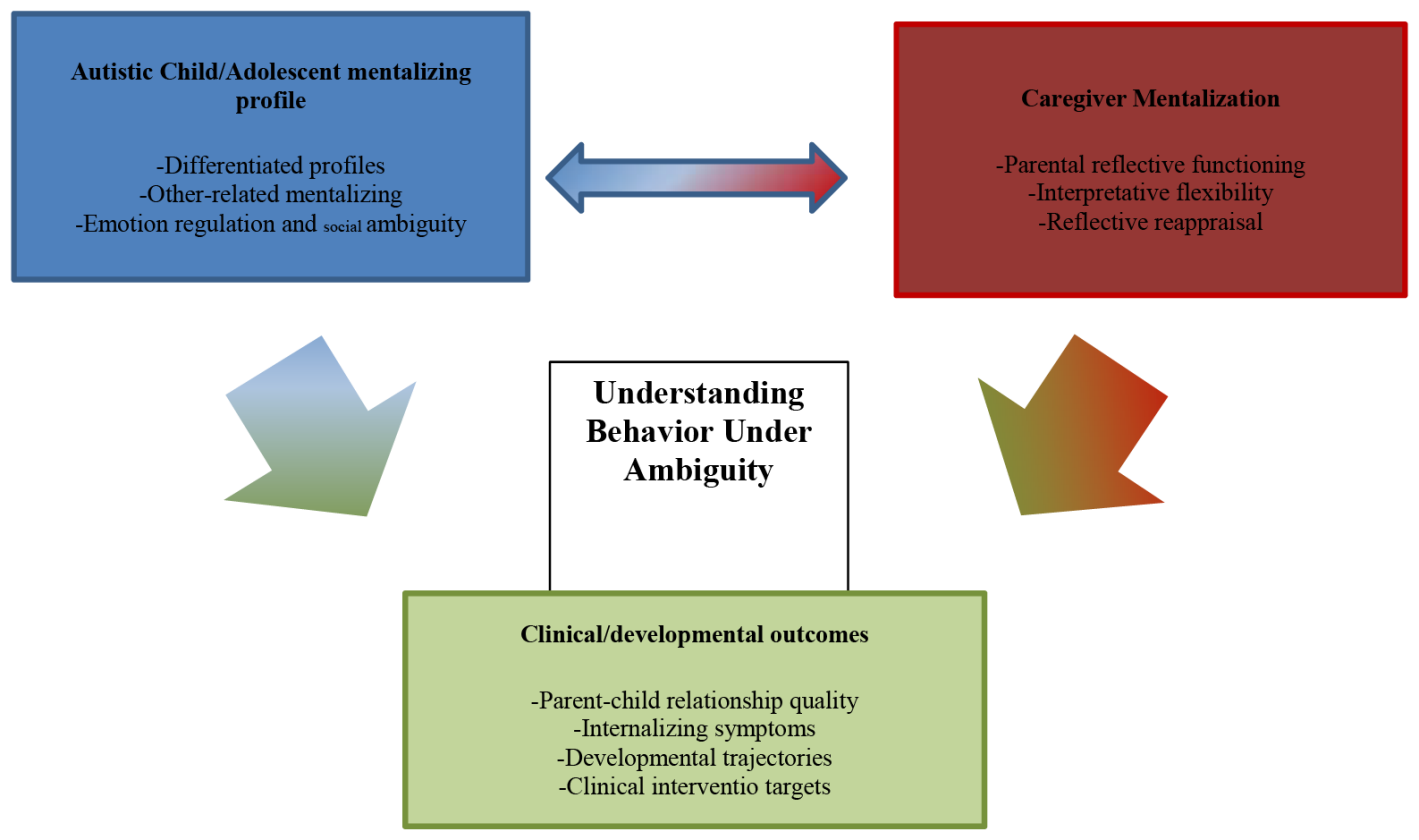
Proposed relational-developmental conceptual framework of mentalization in autism. Mentalization is conceptualized as a differentiated and context-sensitive process involving both autistic children/adolescents and caregiver mentalization. Interactions between child mentalizing profiles and parental reflective functioning may facilitate or constrain shared meaning-making under conditions of social and emotional ambiguity, with potential implications for parent–child relationship quality, internalizing symptoms, developmental processes, and clinical intervention targets. Solid elements are informed by the empirical studies reviewed, whereas the integration of these elements and their proposed reciprocal relationships represents a theory-informed hypothesis intended to guide future research.

From a developmental perspective, the available longitudinal evidence provides preliminary support for a transactional view of mentalization in autism. The bidirectional associations between parental reflective functioning and child behavioral problems suggest that caregiver mentalization and child functioning may influence each other over time (17). Although these findings derive from a relatively short-term longitudinal design, they move the field beyond static models of impairment and toward a dynamic understanding of mentalization as a process shaped through ongoing interactions between children and caregivers. Mentalization may therefore be best understood as a system continuously influenced by the interplay between child characteristics, caregiver responses, and environmental demands. These individual, relational, and developmental processes are integrated in the proposed conceptual framework shown in **Figure 1**, which is intended to organize the available evidence and generate hypotheses for future research rather than represent an empirically validated model.

This relational and developmental framework has direct clinical implications. Mentalization-based and mentalization-informed interventions appear particularly relevant when they target caregiver-child processes, rather than focusing exclusively on the child’s social-cognitive skills. The studies reviewed suggest that caregiver-oriented interventions may improve parental reflective functioning, cognitive reappraisal, self-efficacy, and inferential style, with child outcomes appearing mainly indirect, particularly through reductions in internalizing symptoms (15, 18, 19). These findings suggest that the clinical value of mentalization-based approaches may lie in strengthening caregivers’ capacity to interpret the child’s and their own thoughts and feelings in a more flexible and less deterministic way, thereby supporting emotion regulation and parent-child relationships.

Enav et al. (18) further suggests that one possible mechanism of change may involve reflective reappraisal, namely the capacity to reinterpret emotionally salient situations through consideration of one’s own and others’ mental states. This is clinically relevant because it links mentalization to emotion regulation, suggesting that mentalization-based interventions may not only modify how caregivers understand the child, but also how they regulate their own emotional responses during challenging interactions. In this sense, caregiver mentalization may function as both an interpretative and regulatory process. Nevertheless, these conclusions should be interpreted cautiously, as much of the available intervention evidence derives from a limited number of studies conducted within the same research lineage. Independent replication will be important to determine the reliability and generalizability of these findings.

Clinical models such as MBT-C offer a valuable framework for adapting mentalization-based work to autistic children and their families. Malberg (23) emphasizes the importance of working with both children and caregivers through a predictable yet flexible therapeutic framework, using scaffolding to support attention control, emotion regulation, explicit mentalizing, and the development of new relational patterns. This model is particularly consistent with the view proposed in the present review, because it does not treat mentalization as a purely verbal or cognitive ability. Rather, it highlights the importance of embodied, affective, and interactional forms of meaning-making, including rhythm, playfulness, joint attention, and the caregiver’s capacity to adapt to the child’s regulatory profile. However, Malberg’s contribution should be understood as a clinical and theoretical model rather than empirical evidence of treatment efficacy.

The current evidence base remains preliminary. Most available intervention studies involve small samples, brief interventions, limited follow-up periods, and non-randomized or pre-post designs. In addition, several studies within the relational and intervention domains originate from the same research group. As a result, apparent convergence across studies should be interpreted cautiously until replicated by independent investigators. Moreover, the literature remains largely focused on caregiver-oriented approaches, while interventions directly targeting mentalization in autistic children and adolescents remain comparatively underdeveloped. This represents an important gap, particularly because adolescence may be a developmental period in which social complexity, self-reflection, peer relationships, and vulnerability to internalizing symptoms become increasingly salient.

An additional limitation concerns the relationship between mentalization and psychopathologicalvulnerability in autism. Although mentalization-based approaches have demonstrated clinical value across several psychiatric conditions, including personality disorders and psychosis, empirical evidence directly examining whether mentalization influences the onset, expression, or treatment of co-occurring psychopathology in autistic individuals remains remarkably scarce. Given the high prevalence of psychiatric comorbidities in autism, this gap represents an important missed opportunity. We propose that the current conceptual fragmentation surrounding mentalization may itself contribute to this lack of evidence, as heterogeneous definitions and operationalizations hinder the identification of shared mechanisms and the evaluation of clinically meaningful outcomes. Consequently, the potential role of mentalization as a transdiagnostic framework for understanding and addressing psychopathological vulnerability in autism may be substantially underestimated. Clarifying the construct of mentalization may therefore be critical for evaluating its clinical relevance beyond social functioning, including its potential contribution to the prevention and treatment of co-occurring psychiatric conditions in autistic individuals. Taken together, these considerations suggest that the major challenge for the field is not only methodological but also conceptual, underscoring the need for a more coherent and operationalized framework to guide both research and clinical practice.

Despite the growing interest in mentalization-informed perspectives, the number of empirical studies directly examining mentalization in autism remains limited. Mentalization is often used interchangeably with ToM, social cognition, metacognition, and reflective functioning, without clear specification of how these constructs overlap or differ. This lack of precision limits comparability across studies and complicates the identification of intervention targets and outcome measures. Rather than treating this fragmentation only as a limitation, it may be useful to view it as evidence that mentalization in autism is genuinely multidimensional. The task for future research is not to impose a single narrow definition, but to organize the construct across clinically meaningful dimensions, including self- versus other-related mentalizing, implicit versus explicit processing, cognitive versus affective components, embodied and verbal forms of mentalizing, and individual versus relational levels.

Future research should therefore pursue three priorities. First, studies should define mentalization more precisely and specify which component of the construct is being assessed. Second, measurement approaches should be better aligned with the multidimensional nature of mentalization, combining task-based, questionnaire-based, observational, and relational measures where appropriate. This may also facilitate the development of assessment tools and outcome measures that are more closely aligned with specific dimensions of mentalization and their proposed clinical targets. Third, intervention studies should use more rigorous designs, including randomized controlled trials, larger samples, longer follow-up periods, and clearly defined primary outcomes. This would help clarify whether mentalization-based interventions can produce sustained changes in caregiver-child dynamics and whether these changes reduce psychopathological vulnerability in autistic children and adolescents.

In conclusion, mentalization in autism should not be reduced to a generalized deficit in reading others’ minds. A broader and more clinically meaningful framework conceptualizes mentalization as a dynamic, relational, and developmentally embedded process through which autistic children and adolescents, caregivers, and clinicians construct shared meaning under conditions of social and emotional ambiguity. Moving beyond a narrow deficit-based model may help the field develop more precise assessments, more targeted and developmentally sensitive interventions, and a deeper understanding of psychopathological vulnerability in autism.

## 5 Limitations

This mini-review has several limitations. First, given its targeted narrative design, the literature search was not systematic and did not follow PRISMA guidelines. In addition, the search relied primarily on a single database (PubMed), which may have resulted in the omission of relevant studies, particularly those indexed in psychology-focused databases, introducing a potential selection bias toward the biomedical literature.

An additional limitation concerns the independence of the available evidence base. Several of the empirical studies reviewed, particularly within the relational and intervention domains, were conducted by the same research group. Consequently, support for the proposed framework should be considered preliminary and requires replication across independent samples and research teams. Conclusions regarding individual mentalizing profiles in autistic children and adolescents currently rely on a single empirical study. Moreover, interpretations concerning self-related mentalizing should be considered preliminary, as they are based on the absence of significant group differences rather than on direct evidence of preserved functioning.

Although the proposed framework adopts a developmental perspective, the available developmental evidence remains limited and derives primarily from a small number of studies conducted over relatively restricted time periods. Therefore, the developmental implications of the framework require further longitudinal investigation.

The heterogeneity of constructs, measures, samples, and study designs limited the possibility of directly comparing findings across studies. Furthermore, no formal quality assessment or risk-of-bias appraisal was conducted, consistent with the narrative design of the review. This limitation is particularly relevant when interpreting intervention findings and efficacy-related claims, as the available studies generally involve small samples, brief follow-up periods, and non-randomized or pre-post designs.

Finally, although this review focused on autistic children and adolescents, several theoretical and clinical implications were drawn from the broader mentalization literature and should therefore be tested empirically within autism-specific developmental samples.
